# Effectiveness and implementation of a lifestyle modification intervention for women with isolated impaired fasting glucose: Study protocol for a hybrid type 2 study in Kerala, India

**DOI:** 10.12688/wellcomeopenres.17631.1

**Published:** 2022-02-15

**Authors:** Elezebeth Mathews, Thirunavukkarasu Sathish, Anjaly Joseph, Bhagieshwari Kodapally, Jissa Vinoda Thulaseedharan, KM Venkat Narayan, Brian Oldenburg, Kavumpurathu Raman Thankappan

**Affiliations:** 1 Department of Public Health and Community Medicine, Central University of Kerala, Kasaragod, Kerala, 671320, India; 2 Population Health Research Institute (PHRI), McMaster University, ON L8L 2X2, Canada; 3 Achutha Menon Centre for Health Science Studies, Sree Chitra Tirunal Institute for Medical Sciences and Technology, Thiruvananthapuram, Kerala, 675011, India; 4 Emory Global Diabetes Research Center, Emory University, Atlanta, Georgia, GA 30322, USA; 5 Baker Heart and Diabetes Institute, Baker Heart and Diabetes Institute, Melbourne, Victoria, Victoria 3004, Australia

**Keywords:** Prediabetes, Isolated impaired fasting glucose, Lifestyle modification, Kerala, Women, RCT

## Abstract

**Background:** Isolated impaired fasting glucose (i-IFG) constitutes a major group in the prediabetic spectrum among Indians, and thus it is imperative to identify effective diabetes prevention strategies. This study aims to evaluate the effects of an intensive community-based lifestyle modification program on regression to normoglycemia among women with i-IFG, compared to a control group at 24 months. The study also aims to evaluate the implementation of the intervention, via both process and implementation outcomes.

**Methods:** We will use a hybrid design (Effectiveness-implementation hybrid type 2 trial) to test the effectiveness and implementation of the lifestyle modification intervention. Effectiveness is evaluated using a randomized controlled trial among 950 overweight or obese women, aged 30 to 60 years, with i-IFG on an oral glucose tolerance test in the Indian state of Kerala. The intervention involves an intensive lifestyle modification program through group and individually mentored sessions using behavioural determinants and behavioural change techniques.  The intervention group will receive the intervention for a period of 12 months and the control group will receive general health advice through a health education booklet. Data on behavioural, clinical, and biochemical measures will be collected using standard methods at 12 and 24 months. The primary outcome will be regression to normoglycemia at 24 months, as defined by the American Diabetes Association criteria.

**Discussion**: This study will provide the first evidence on the effects of lifestyle interventions on regression to normoglycemia in people with i-IFG among Indians.

**CTRI registration:** CTRI/2021/07/035289 (30/07/2021)

## Introduction

Type 2 diabetes mellitus (T2DM), currently affects almost 537 million adults worldwide with a projected increase of nearly 50% by 2045 ^1^. Low- and middle-income countries (LMICs) such as India are disproportionately affected, with the large majority (81%) of people with T2DM living in these countries ^1^. India has the second-largest number of people (74 million) living with T2DM and this is projected to increase to 125 million people by 2045 ^1^. In addition, T2DM poses a significant economic burden in LMICs, affecting not just the health care system but also individuals and families with increased out-of-pocket spending for diabetes care ^2^. Therefore, much importance has been given to diabetes prevention, with a greater research focus on individuals who are at high risk for diabetes.

Prediabetes, a high-risk metabolic state for diabetes, comprises impaired fasting glucose (IFG) and impaired glucose tolerance (IGT), wherein the glucose levels are higher than those considered to be normal, but lower than the threshold for T2DM ^3^. A recent review of 19 studies globally showed that among those with prediabetes, the average proportions of isolated IFG (i-IFG), isolated IGT (i-IGT), and combined IFG and IGT were 43.9%, 41.0%, and 13.5%, respectively, in Caucasians, and 29.2%, 49.4%, and 18.2%, respectively, in Asians ^4^. A large-scale study in 15 states of India showed that i-IFG is the most common form of prediabetes among adults, with a prevalence of 21% ^5^. On average, nearly 5–10% of people with prediabetes develop T2DM every year, although the progression rate varies by population characteristics (including ethnicity) and the definition of prediabetes ^6^. With no effective intervention, nearly 70% of people with prediabetes would eventually progress to develop T2DM ^6^.

Lifestyle modification is recognized as an effective non-pharmacologic intervention to prevent or delay the onset of diabetes among individuals with prediabetes ^7^. Most landmark lifestyle modification randomized controlled trials (RCTs) for diabetes prevention have been conducted in individuals with IGT ^8^, with very limited evidence in the i-IFG group. Lifestyle modification in the trials for those with IGT have focused mainly on weight loss and promoting physical activity, although the intervention intensity, goals, and delivery methods have varied widely between the trials. These trials have shown that diabetes incidence could be reduced between 28.5% and 58% in the intervention group compared with the control group ^8^. More importantly, a clinically meaningful reduction in diabetes incidence with lifestyle modification has been shown to persist in those with IGT even after 30 years of follow-up ^9^.

Only a few RCTs have evaluated the effects of lifestyle modification on diabetes prevention in people with i-IFG ^10^. RCTs conducted among 641 overweight Japanese ^11^ and 578 overweight Indian adults ^12^ showed hazard ratios of 1.17 (95% CI 0.50-2.74, n=379) and 0.88 (95% CI 0.43-1.20, n=166) respectively, in those with i-IFG at 3 years. Similarly, a trial among 880 adults with prediabetes in the UK showed a hazard ratio of 0.52 (95% CI 0.15-1.83, n=108) at 3 years in those with i-IFG ^13^. The Kerala Diabetes Prevention Program (K-DPP) from India showed a relative risk of 0.95 (95% CI 0.68, 1.33) in people with i-IFG (n=579) at 2 years among 1007 high-risk individuals ^14^. However, the above-mentioned findings were from the sub-group analyses, which are constrained by small sample sizes, a limited number of events, and confounding ^15^.

While preventing the progression of prediabetes to diabetes is important, regression to normoglycemia is also essential to achieve, even if transient, as this has been shown to be significantly associated with reduction in the development of diabetes ^10^. To our knowledge, there are no data on regression to normoglycemia with lifestyle modification in the i-IFG group among Indians, an ethnic group with i-IFG being the most common prediabetes phenotype ^5, 14^. In addition to being at high risk for diabetes, individuals with i-IFG are at an increased risk of developing micro- and macro-vascular complications, and of all-cause mortality ^6^.

This study aims to evaluate the effects of an intensive community-based lifestyle modification program on regression to normoglycemia among women with i-IFG compared to a control group at 24 months. We will also evaluate the effects of the intervention on improving cardiometabolic risk factors. The study will use the RE-AIM framework to evaluate Reach, Adoption, Implementation and Maintenance ^16^ and the intervention fidelity using process measures.

This two-arm parallel group randomized controlled trial targets women as the behavioural risk factors of overweight or obesity and physical inactivity are generally higher among women, compared to men, in the Indian context ^17^ and the intervention outcomes reported so far are poorer among women ^12^ in India.

## Methods

### Study design and setting

The study uses a hybrid design (Hybrid type 2) ^18^ that tests the effectiveness and implementation of a lifestyle modification program. A randomized controlled trial will evaluate the effectiveness of the intervention, and the intervention implementation and fidelity will be evaluated using implementation outcomes and process measures, respectively.

The study was registered with Clinical Trials Registry, India (CTRI/2021/07/035289, on 30 ^th^ July 2021). The trial will be reported in accordance with the Consolidated Standards of Reporting Trials (CONSORT) guidelines ^19^.

**Figure 1.**
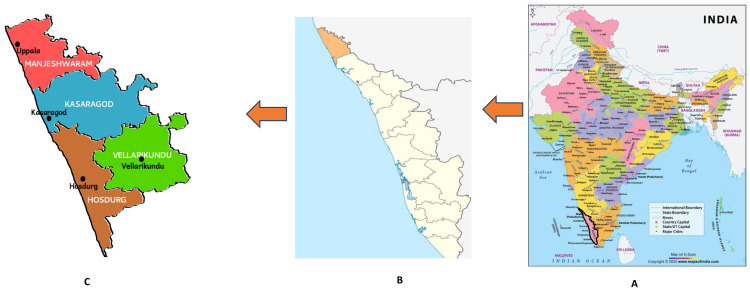
Map of the study area. **A.** India political. **B.** Kasaragod district (shaded) in the State of Kerala. **C**. Taluks of Kasaragod district.

The study will be conducted in Kasaragod district of Kerala ( Figure 1). Kerala, the southernmost Indian state, has a higher prevalence of T2DM (nearly 20%) and a greater burden of several cardiometabolic risk factors than most other Indian states ^17^. Further, the state is in the most advanced stage of epidemiological transition in the country ^20^ and said to be a harbinger of the future burden of diabetes and other chronic diseases in India ^20^. Thus, Kerala provides an ideal place to implement and evaluate lifestyle modification programs.

**Figure 2.**
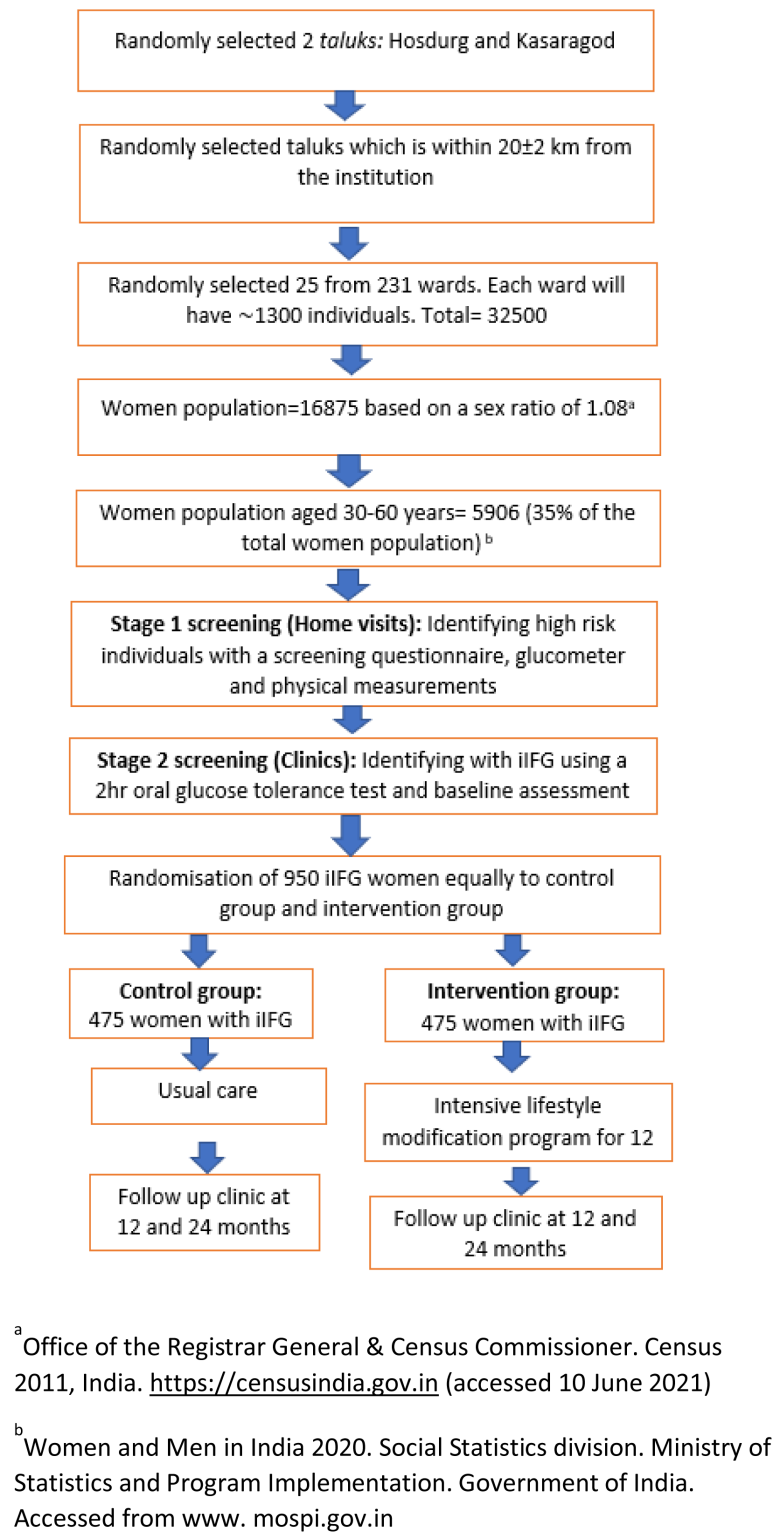
CONSORT diagram of the trial.

Figure 2 shows the CONSORT diagram of the trial. Kasaragod is the northernmost district of Kerala, with a population of 1,307,375, a sex ratio of 1079 women for every 1000 men, and a literacy rate of 90% ^21 ^. Kasaragod has four *taluks* (sub-district) with 777 wards (lowest administrative division with approximately 1300 individuals in each ward) ^21^. From these four *taluks*, two were randomly selected, namely Hosdurg *taluk* and Kasaragod *taluk*. There are 465 wards in these two taluks, amongst which those wards within 20km distance from the institute were included (269 wards) considering the logistics and feasibility. Out of 269 wards, 25 wards with at least 1000 individuals in each ward were randomly selected. From these selected wards, individuals with i-IFG will be identified and recruited to the trial with a minimum of 23 participants per ward.

### Study participants

***Inclusion criteria*:** 1) Women aged 30–60 years; 2) overweight or obese (waist circumference ≥80 cm) ^22^; 3) no prior history of diabetes; 4) not taking any glucose-lowering medications; 5) no prior history of gestational diabetes mellitus (GDM); 6) able to read, write and speak Malayalam, the local language; 7) consents to participate in the trial; and 8) diagnosed with i-IFG (fasting plasma glucose [FPG] 5.6-6.9 mmol/l and 2-hr plasma glucose [2-hr PG] <7.8 mmol/l) on a 2-hr oral glucose tolerance test (OGTT) ^3^.

***Exclusion criteria*:** 1) Women with known T2DM; 2) having GDM; 3) prior history of GDM; 4) breastfeeding women; 5) having major chronic illnesses including mental disorders, that are likely to impede consenting and participation in the intervention program; 6) taking medications that could alter glucose metabolism (e.g., corticosteroids); and 7) diagnosed with normoglycemia, IGT, and T2DM, as per the American Diabetes Association criteria ^3^ on the OGTT.

### Sample size and randomization

We assumed that the cumulative incidence of regression to normoglycemia at two years would be 18% in the control group (based on unpublished data from the K-DPP trial) and that there would be a 50% relative risk in conversion to normoglycemia with the intervention. The sample size required in each study group was 475 with a type 1 error of 5%, at least 80% power, a contamination rate of 15%, and a 10% loss to follow-up. The sample size required with same estimates and 90% power in each group is 625, and will be recruited, if logistically feasible.

The estimated sample size of 950 participants will be randomly allocated in a 1:1 ratio to the control group and the intervention group using a computer-generated randomization sequence using Microsoft excel by an independent statistician not involved in the trial. The independent statistician will generate the randomized sequence and allocate the individuals to the randomized groups. The participants and the Principal Investigator (EM) will be blinded to the allocation sequence until informed written consent for study participation is obtained. The outcome assessors, data entry personnel and statisticians who analyse the data will be blinded throughout the study as they are not part of the team that delivers intervention or manage the project.

This study uses individual randomization in order to avoid the methodological challenges of cluster randomization, including the clustering effect on statistical power and selection bias, possibility of imbalanced study groups, and the dilution effect ^23^. Trial conduct solutions will be put in place to address potential contamination, if any, between the study groups with individual randomization. This include using different staff for each group, education of the participants against contamination ^23^, and getting signed nondisclosure agreement from the intervention participants regarding the type of intervention being received. Furthermore, analytical methods that adjust for contamination will also be used. We will also quantify the contamination using a treatment fidelity framework based on the criteria advocated by the Behaviour Change Consortium ^24^ and NICE guidance on behaviour change ^25^.

### Recruitment of participants and data collection

Screening and recruitment of participants and data collection will be conducted in 2 stages as described below:

***Stage 1: Home visits*.** All women in the age group of 30–60 years will be selected from the voters' list of the selected wards. Eligibility for participation in the study will be ascertained during home visits using a screening questionnaire (includes demographics and eligibility criteria) by trained staff. Only one participant per household (randomly selected) will be screened. Those who meet the eligibility criteria and provide consent for study participation will be screened with a point-of-care glucometer (OneTouch Select Plus, LifeScan Inc.) and their waist circumference will be measured using SECA 201 ergonomic retractable tape with using a standard protocol ^26^. Those who are overweight or obese (waist circumference ≥80 cm) ^22^ and with a random capillary blood glucose of >110 mg/dl ^27^ will be invited to undergo a 2-hr OGTT in clinics organized in their neighbourhoods. These individuals will be considered to be at high risk for having prediabetes or diabetes.

***Stage 2: Clinics*.** The high-risk individuals will attend clinics organized in their neighbourhoods, where they will undergo a 2-hr OGTT, body composition, anthropometric, and blood pressure measurements, and interviews to complete the questionnaires. Those diagnosed with T2DM on the OGTT will be referred to the nearby health facility for treatment and care. Body fat composition will be assessed using TANITA DC 360 (pole). Blood pressure will be measured using an OMRON HEM-7120 blood pressure monitor using the standard protocol ^26^.

The weight and height will be measured using SECA 813 flat scale and SECA 213 stadiometer, respectively, with a standard protocol ^26^. Blood samples will be collected in a fasting state (8–10 hours of fasting) and will be centrifuged within 30 mins after collection and transported to a lab accredited by the National Accreditation Board for Testing and Calibration Laboratories (NABL) ^28^. Questionnaires will be administered by trained staff to collect information on demographics, diet ^29^, physical activity ^26^, tobacco use ^26^, alcohol use ^26^, diabetes knowledge ^30^, prediabetes knowledge ^31^, health-related quality of life (HRQoL) ^32^; self-efficacy for managing chronic diseases ^33^, risk perception for diabetes ^34^, social support ^35^, and sleep hygiene ^36^ using standardized questionnaires ( Table 1). Those who are diagnosed with diabetes at 12 ^th^ and 24 ^th^ month assessment will be referred to the nearby health facility for treatment and care, and will continue to participate in the trial. All assessments done at the baseline will be repeated at 12 months and 24 months.

**Table 1.** Outcomes, measurement tools, and data collection time points.

Outcome measures	Variable	Tools/tests used	Baseline	Regular assessment	12 months	24 months
Primary outcome for effectiveness						
Regression of i-IFG to normoglycemia at 12 and 24 months	Fasting plasma glucose and 2-hr post-load plasma glucose	OGTT in a NABL accredited laboratory ^28^	✓		✓	✓
Secondary outcomes for effectiveness						
Incidence of diabetes	Fasting plasma glucose, 30 minutes and 2-hr post- load plasma glucose, HbA1c	NABL accredited laboratory ^28^	✓		✓	✓
Insulin sensitivity	Fasting insulin, 2-h insulin, Homa IR	NABL accredited laboratory ^28^	✓		✓	✓
Beta cell function	Fasting insulin, 2hr insulin, Homa B		✓		✓	✓
Lipid profile	Total cholesterol, triglycerides, HDL-cholesterol, and LDL-cholesterol	NABL accredited laboratory ^28^	✓		✓	✓
Liver function tests	Aspartate aminotransferase (AST), alanine aminotransferase (ALT), gamma-glutamyl transferase (GGT)	NABL accredited laboratory ^28^	✓		✓	✓
Blood Pressure	Systolic and diastolic blood pressure	OMRON electronic blood pressure monitor	✓		✓	✓
Anthropometrics	a. Weight b. Height c. Waist circumference	a. SECA weighing scale b. SECA stadiometer c. SECA non-elastic tape	✓		✓	✓
Body composition measures	a. Fat percent b. Muscle mass	TANITA body fat analyser	✓		✓	✓
Psychosocial variables	a. Diabetes knowledge b. Knowledge on prediabetes c. Health-related quality of life d. Self-efficacy/self-empowerment e. Risk-perception for diabetes f. Social support g. Sleep Hygiene	a. 24 Item Diabetes Knowledge Questionnaire ^30^ b. Prediabetes Knowledge Questionnaire ^31^ c. WHO QoL BREF scale ^32^ d. Health Lifestyle and Personal Control Questionnaire ^33^ e. Scale by Hivert *et al.* ^34^ f. Scale by Sarason *et al.* ^35^ g. Sleep Hygiene Index scale by Mastin *et al.* ^36^	✓		✓	✓
Behavioural outcomes for effectiveness and implementation						
	a. Diet b. Physical activity and sedentary behaviour c. Tobacco use d. Alcohol use	a. Food frequency questionnaire ^25^ b. Global Physical Activity Questionnaire ^26^ c. WHO STEPS questionnaire ^26^ d. WHO STEPS questionnaire ^26^	✓		✓	✓
Implementation outcomes (at community, provider and beneficiary levels)						
	a. Reach a1. Participants approached a2. Frequency of contact with the *panchayat* b. Effectiveness b1. Improved knowledge on T2DM prevention b2. Risk perception b3. Self-efficacy b4. Social Support c. Adoption c1 Individuals attending the sessions (Number) c2. Individuals who set goals c3 Individuals achieving the behavioural targets c4. Change in knowledge on diabetes and its risk factors and behavioural targets d. Implementation d1. Peer mentor trainings (quality) d2. Participants group sessions (quality) d3. Sessions conducted d4. Peer mentor selection d5. Support received by peer mentors e. Maintenance e 1. Individuals achieving behavioural targets	a1. Database on recruitment a2. Database on meetings b1. 24 Item Diabetes Knowledge Questionnaire ^30^ b.2. Risk perception Scale ^34^ b.3 Health Lifestyle and Personal Control Questionnaire ^33^ b4. Social Support scale ^35^ c1. Participant attendance data c2. Participants feedback report c3. Behavioural change report ^24, 25^ c4. Pre and post knowledge change evaluation report (Participant) d1. Peer mentors feedback report, Pre and post knowledge change evaluation report (Peer Mentors) d2. Participants feedback report, d3. Session attendance data d4. 1. Peer mentors characteristics d5. Participant feedback report e1. Behavioural assessment data ^24, 25^	✓ ✓ ✓ ✓	✓ ✓ ✓ ✓ ✓ ✓ ✓ ✓ ✓ ✓ ✓	✓ ✓ ✓ ✓	✓ ✓ ✓ ✓ ✓

OGTT- Oral Glucose Tolerance Test, NABL-National Accreditation Board for Testing and Calibration Laboratories, WHO QoL BREF- World Health Organization Quality of Life , HDL- High density lipoprotein, LDL- Low density lipoprotein

Staff conducting physical measurements and administering questionnaires at baseline and follow-up visits will be blinded to participants’ group allocation. Intervention staff will not be engaged in the data collection process and the statistician engaged in data analysis will be blinded to the group allocation.

All study materials can be found in the *Extended data* ^37^.

### Outcomes

The primary outcome for the effectiveness study will be normoglycemia, defined as FPG<5.6 mmol/l and 2-hr PG<7.6 mmol/l, at 24 months ^3^. Secondary outcomes will include incidence of T2DM (fasting plasma glucose ≥ 7.0 mmol/l or 2hr PG ≥ 11.1 mmol/l or HbA1c ≥6.5%), insulin sensitivity, beta cell function, weight, body mass index, waist circumference, FPG, 2-hr PG, blood pressure, body composition measures (fat percent and muscle mass) and psychosocial variables (health-related quality of life, self-efficacy/self-empowerment, risk-perception for diabetes, social support and sleep hygiene). Implementation outcomes include reach, effectiveness, adoption, implementation and maintenance of the intervention ^16^.

### Study groups

***Control group*.** Participants in the control group will receive a health information leaflet at baseline in local language ( *Malayalam)* on strategies for diabetes prevention. No further engagement will be there in the control group apart from follow-up assessments at 12 and 24 months.

***Intervention group*.** The study participants in the intervention group will receive an intensive lifestyle modification program for a period of 12 months through group and individually mentored sessions. The intervention will include behavioural determinants such as self-efficacy, risk perception, social support, and behavioural change techniques, including knowledge enhancement, self-monitoring, goal setting and review, and peer support ^38^ ( Table 2). The intervention will be intense as the engagement is not just limited to group sessions, but also involves individualized support through peer mentors and individualized instructions through a mobile application, specifically made for this trial.

**Table 2.** Program goals, theory-based methods, and strategies (Adapted from the Kerala Diabetes Prevention Program) [Source: Elezebeth et al. Cultural adaptation of a peer-led lifestyle intervention program for diabetes prevention in India: the Kerala Diabetes Prevention Program (K-DPP) ^38^]

Program Goals	Personal learning and Social environmental change objectives	Determinants based on theory and evidence	Behaviour change techniques	Feasible and culturally acceptable strategies to enhance engagement and implementation
a. Weight loss by 5–7% ^6^ b. Reduction in waist circumference by ≥4 cm ^22^ c. Reduction in body mass index ^6^ by ≥0.5 kg/m ^2^ d. Increased consumption of fruit and vegetables (>5 servings/day) ^39^ e. Increased physical activity through walking, exercise, and culturally appropriate activities (>150 minutes/ week) ^40^ f. Improved sleep hygiene ^41^	**Personal learning objectives** • Increase in awareness of risk factors of T2DM • Improve risk perception on T2DM • Improve self-efficacy in making lifestyle changes **Social Environment change** **objectives** • Enhance peer support for behaviour change • Enhance household/family support for behaviour change • Facilitate opportunities for healthy lifestyle in collaboration with group members • Empowerment for diabetes prevention	• Risk perception • Self-efficacy • Social support • Availability and accessibility of facilities for physical activity and healthy food options	• Provide information on the risk factors of T2DM • Provide information on the dietary and physical activity targets for individuals. • Self-monitoring • Goal setting and goal review with emphasis on participants and family member outcomes • Peer support • Social and practical support from family, neighbourhood members and community organizations (Panchayats) • Engage and empower family and group members to increase availability and accessibility of healthy food options and physical activity options	**Individual-level** • Educational sessions that focus on ‘modifiable’ determinants of risk on diabetes. • Sessions scheduled in local neighbourhoods (e.g. a reading room or *Kudumbashree’s meeting* *rooms*) according to work, family and other cultural needs of participants **Interpersonal-level (family)** • Group-based delivery • Inclusion of family members in the sessions • Enabling ongoing peer and social support, with family members and friends of participants • Kitchen garden training to facilitate vegetable consumption and increase enjoyable physical activity • Forming walking groups or other activity groups such as yoga or aerobic dance groups as appropriate.

***Theory of intervention*.** The intervention program objectives, theory-based methods, and strategies for intervention engagement and implementation are given in Table 3. Briefly, the intervention was adapted from a successfully conducted lifestyle-based diabetes prevention trial in Kerala, the K-DPP ^38^ Program objectives will be achieved through “personal learning” and “environmental change” using evidence-based behaviour change techniques with strategies targeting participants at individual, interpersonal and community levels.

**Table 3.** Intervention sessions schedule.

Session	Individual/ Group	Theme	Activity (AV aid)	Duration	Facilitator
1	G	1.1 Introduce the project 1.2 Rapport building 1.3 Knowledge enhancement	1.1.1 Introduction about the project, requirements and commitments 1.2.1 Building rapport with the participants 1.2.2 Identification of peer mentor 1.3.1 Discuss about diabetes and prediabetes (IFG & IGT) (Flipchart & Mobile based application) 1.3.2 Discuss about risk factors of diabetes (Flipchart & Mobile based application)	20 Min 15 Min 30 Min 20 Min 20 Min	Research Team
2	G	2.1 Risk factor Modification- Unhealthy break/>diet 2.2 Self-monitoring of dietary intake 2.3 Knowledge enhancement on dietary allowances and recommendation for fat/sugar/ vegetables/fruits/salt/rice) 2.4 Goal setting 2.5 Diet monitoring	2.2.1 Self-monitoring work sheet (Flipchart & participant workbook) 2.3.1 Education on healthy diet practices [session+ activity] (Flip chart & Mobile based application) 2.4.1 Individualised goal setting (Flipchart & participant workbook) 2.5.1 Personalized diet planning tool using an interactive mobile application platform.	20 Min 20 Min 20 Min 15 Min	Research Team
3	I	3.1. Diet goal monitoring and revisiting the goals	3.1.1. Goal monitoring (participant workbook) 3.1.2. Identification of barriers 3.1.3. Goal resetting (if needed)	10 Min 10 Min 10 Min	Peer mentor
4	G	4.1 Demonstration of healthy diet option (Salad preparation) 4.2 Physical activity promotion 4.3 Self-monitoring 4.4 Knowledge enhancement on strategies to improve PA 4.5 Goal Setting	4.1.1 Demonstrate the preparation of healthy diet (salad) 4.2.1 Discuss on physical activity and its importance (Flip chart) 4.3.1 PA self-monitoring worksheet (participant workbook) 4.4.1 Discuss on the types of physical activities that are culturally appropriate and feasible (Flipchart & participant workbook) 4.5.1 Individualised goal setting (Participant workbook)	20 Min 15 Min 15 Min 25 Min 15 Min per participant	Research Team
5	I	5.1 PA goal monitoring and revisiting the goals 5.2 Diet goal monitoring and revisiting the goals	5.1.1 Goal monitoring (Participant workbook) 5.1.2 Identification of barriers 5.1.3 Goal resetting (if needed) 5.2.1 Goal monitoring (Participant workbook) 5.2.2 Identification of barriers 5.2.3 Goal resetting (if needed)	30 Min per participant	Peer mentor
6	1	6.1 PA goal monitoring and revisiting the goals 6.2 Diet goal monitoring and revisiting the goals	6.1.1 Goal monitoring (Participant workbook) 6.1.2 Identification of barriers 6.1.3 Goal resetting (if needed) 6.2.1 Goal monitoring (Participant workbook) 6.2.2 Identification of barriers 6.2.3 Goal resetting (if needed)	30 Min per participant	Peer mentor
7	G	7.1 Knowledge enhancement - Effect of stress on diabetes and other chronic diseases 7.2 Strategies for Stress Management 7.3 Strategies to enhance Sleep hygiene	7.1.1 Awareness on impact of stress and importance of managing it (Flipchart & m health app) 7.2.1 Identification of Stress factors (Flipchart) 7.2.2 Demonstration of various stress management techniques (Breathing exercise and yoga) 7.3.1 Educate about the importance of maintaining sleep hygiene (Flipchart)	20 Min 20 Min 10 Min 15 Min	Research Team
8	I	8.1 PA goal monitoring and revisiting the goals 8.2 Diet goal monitoring and revisiting the goals	8.1.1 Goal monitoring (Participant workbook) 8.1.2 Identification of barriers 8.1.3 Goal resetting (if needed) 8.2.1 Goal monitoring (Participant workbook) 8.2.2 Identification of barriers 8.2.3 Goal resetting (if needed)	30 Min per participant	Peer mentor
9	I	9.1 PA goal monitoring and revisiting the goals 9.2 Diet goal monitoring and revisiting the goals	9.1.1 Goal monitoring (Participant workbook) 9.1.2 Identification of barriers 9.1.3 Goal resetting (if needed) 9.2.1 Goal monitoring (Participant workbook) 9.2.2 Identification of barriers 9.2.3 Goal resetting (if needed)	30 Min per participant	Peer mentor
10	G	10.1 Tobacco and alcohol cessation	10.1.1 Create awareness on impact of alcohol and tobacco in the development of T2DM 10.1.2 Refer the participants who use tobacco and alcohol to cessation clinics (if needed)	20 Min	Research Team
11	I	11.1 PA goal monitoring and revisiting the goals 11.2 Diet goal monitoring and revisiting the goals	11.1.1 Goal monitoring (Participant workbook) 11.1.2 Identification of barriers 11.1.3 Goal resetting (if needed) 11.2.1 Goal monitoring (Participant workbook) 11.2.2 Identification of barriers 11.2.3 Goal resetting (if needed)	30 Min per participant	Peer mentor
12	I	12.1 PA goal monitoring and revisiting the goals 12.2 Diet goal monitoring and revisiting the goals	12.1.1 Goal monitoring (Participant workbook) 12.1.2 Identification of barriers 12.1.3 Goal resetting (if needed) 12.2.1 Goal monitoring (Participant workbook) 12.2.2 Identification of barriers 12.2.3 Goal resetting (if needed)	30 Min per participant	Peer mentor

### Intervention content

**Figure 3.**
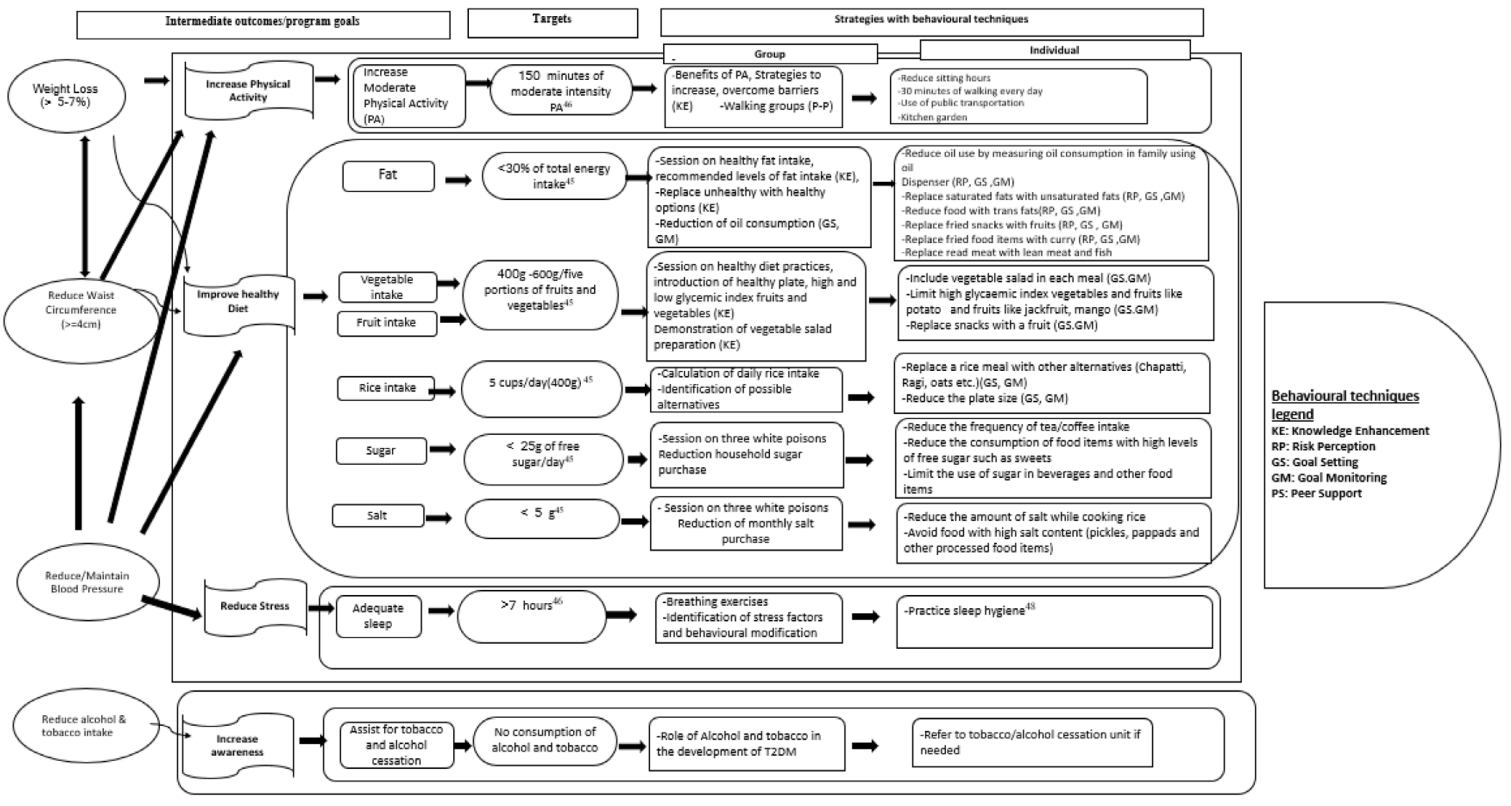
Thematic representation of the program goals, targets and individual-group tailored strategies using behaviour change techniques

Figure 3 shows a thematic representation of the program goals with the targets and the strategies adopted at individual and group level using the behaviour techniques. The intervention will focus primarily on key behavioural risk factors, including unhealthy diet, physical inactivity, tobacco use, alcohol use, and sleep. Based on the dietary recommendations for the prevention of T2DM ^39^ and pertinent research findings among individuals with IFG, the dietary intervention will include consumption of a low-calorie diet (~1500 calories per day i.e., 500 calories lower than the daily requirement for women) ^39, 42– 44 ^and consuming food with low glycaemic index. Other dietary recommendations include changing the quality of dietary fat from using saturated to unsaturated, increasing the intake of whole grains and foods rich in fibre and decreasing the intake of sugar-sweetened beverages, sweets, and highly processed products ^45– 47^. The dietary goals are: <30% of total energy intake from fat, 400–600 grams of fruit and vegetable intake a day, 5 cups (400 gms of cooked rice intake per day), <25 gms of free sugar intake a day, and <5 gms of salt intake per day ^45^. The adherence to diet will be assessed every month using a 24-hour dietary recall ^48^. Other measures of intervention goal include increasing physical activity to the recommended levels of at least 150 minutes of moderate-vigorous physical activity per week ^40^. 7–9 hours of sleep at night ^41, 49^, and no use of alcohol and tobacco.

### Intervention delivery

The intervention will be delivered through 12 sessions (individual and group based), one session per month, over a period of 1 year ( Table 3). Intervention delivery will be supported using pretested and piloted educational materials such as flip charts for group-based sessions and a mobile based application for individual sessions. The mobile application will also serve as an interactive platform for a personalized diet planning and reporting.

The group sessions (45–60 minutes duration) will be organised in the participants’ neighbourhood, mostly in homes or local health centres, delivered by the research team (postgraduates in public health/ social work degree) in the initial phase, followed by trained volunteers as “peer mentors”. Peer mentor, a group nominated volunteer, will undergo a five-day capacity building training program to guide and assist the participants in making realistic goals for lifestyle modification with the support of the extended community stakeholders.

### Evaluation framework

**Figure 4.**
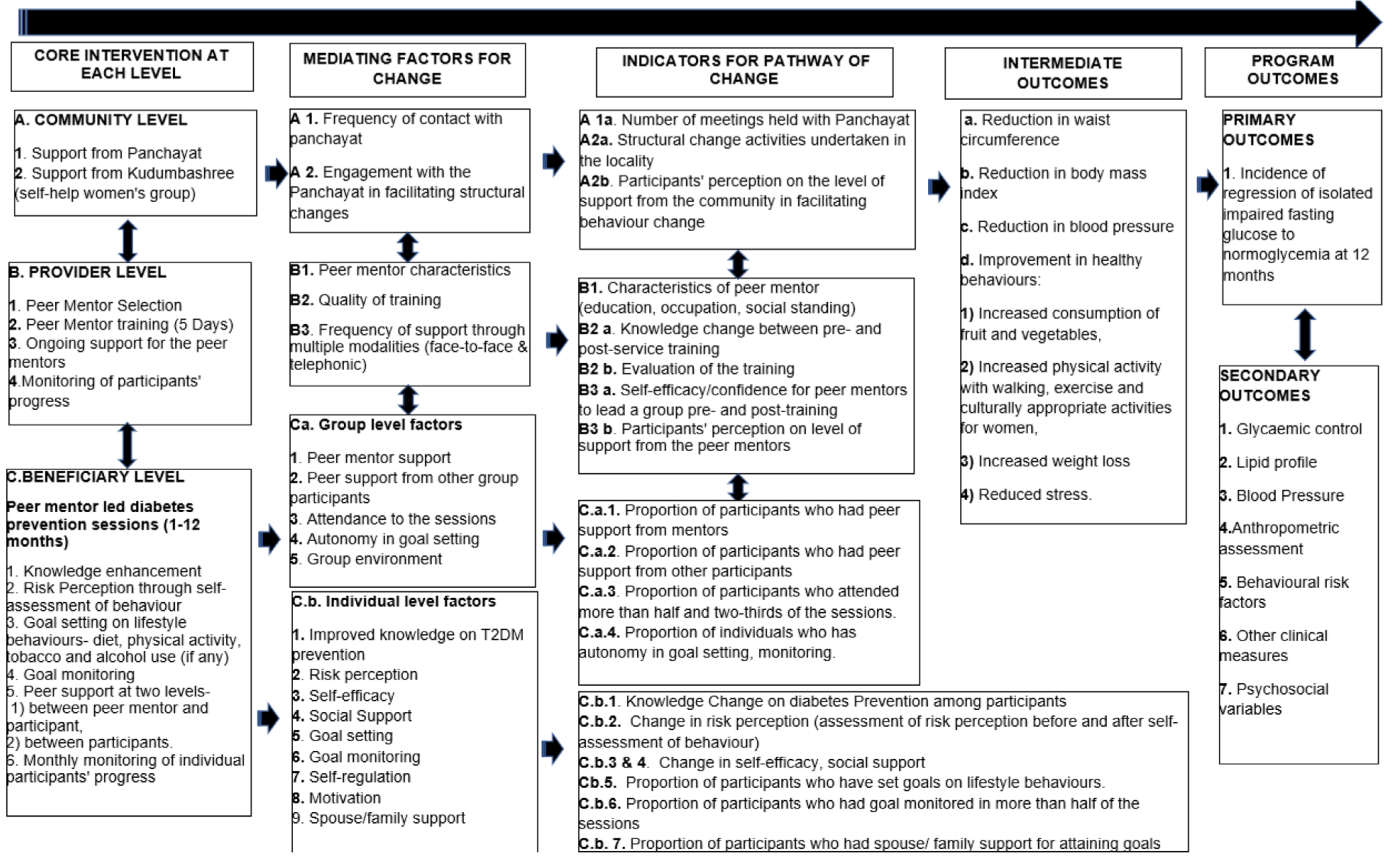
Theory of change Framework.

In addition to the baseline, 12th month and 24 ^th^ month assessment for the primary and secondary outcomes, process evaluation of core interventions at community, peer mentor and participant levels will be undertaken ( Figure 4). Evaluation process will be facilitated through regular monitoring via participant feedback report, peer mentor feedback report, feedback on quality of training, pre- and post-training knowledge evaluation and other interactions (mobile app/telephonic contact). 

### Data analysis

Data will be collected and entered using a data entry template in an ODK platform by the data collectors and managed in a cloud-based server which will only be accessed by the Principal Investigator (EM). Subsequent to the data collection, the data will be used only using a participant identity number and all the personal identifiers will be masked. Only deidentified data will be shared with other investigators, if required. Data quality will be ensured during data collection and data analysis. During the data collection, the research team will verify the data for missing values and if present, will be rectified then and there. Furthermore, randomly identified 5% of the participants data will be verified with the participants through telephone for quality check. The data from the ODK platform will be exported to SPSS and data cleaning will be done manually. Any outliers or implausible values will be identified and will be checked with participants over phone, if necessary.

The analysis will follow the intention-to-treat (ITT) principle. Characteristics of the participants at baseline will be compared between study groups using descriptive statistics. The primary outcome will be analysed using the generalized estimating equations (GEE) with an appropriate working correlation structure and a binomial family with 'log' link function to estimate the relative risk (and 95% confidence interval [CI], p value). Standard errors will be based on Huber-White sandwich estimator, which will provide valid CIs even in case of misspecification of the correlation structure ^50^. For secondary outcomes, continuous variables will be analysed using mixed-effects linear regression models, which will include all available data at baseline, 12 and 24 months. Study group (intervention vs. control), timepoint (follow-up vs. baseline) and a study group-by-time point interaction will be specified as fixed effects. Random effects will be specified for wards, to account for the clustered study design, and for participants, to account for correlation between the repeated measurements on the same individual. Categorical variables will be analyzed using log-binomial models. All p values reported will be 2 tailed, and a p of 0.05 will be considered statistically significant. Analyses will be performed with Stata version 14.2 (StataCorp LP, College Station, Texas, USA). 

### Ethics approval

The study was approved by the Institutional Human Ethics Committee of the Central University of Kerala (CUK/IHEC/2019/034_A, 21 ^st^ November 2019). Written informed consent will be obtained from all study participants. The risk from the intervention to the participants is anticipated to be negligible as the intervention involves only lifestyle modification and no pharmacological drugs. Data safety monitoring will be done by the research team.

## Discussion

This paper describes the protocol for an intense lifestyle intervention program among women with i-IFG to facilitate regression to normoglycemia at 24 months. This study will provide the first evidence on the effects of lifestyle intervention in regressing i-IFG to normoglycemia among Indians. The findings of the study will be disseminated through public engagement, reports and publications.

### Strengths and limitations

This is one of the first studies globally to evaluate the effects of a novel lifestyle intervention among women with i-IFG. Further, the community-based nature of the intervention would facilitate future sustainability and scalability in India and other similar settings. However, since the study population comprises only women, the findings cannot be generalized to men.

### Trial status

The trial is currently in the screening phase.

## Data availability

### Underlying data

No underlying data are associated with this article.

### Extended data

OSF: Randomized Controlled Trial on lifestyle modification intervention for women with isolated impaired fasting glucose. https://doi.org/10.17605/OSF.IO/8K9XJ ^37^.

This project contains the following extended data:

- Participant information sheet (screening).pdf
- Informed consent form screening.pdf
- Screening tool.pdf
- Participant information sheet for the trial (English version).pdf
- Informed consent form for trial.pdf
- Baseline assessment.pdf
- 12 ^th^ month assessment.pdf
- 24 ^th^ month assessment.pdf
- Process evaluation tools- participants.pdf
- Process evaluation tools - peer mentor.pdf
- Health Information leaflet Control.pdf (for control participants)

Data are available under the terms of the Creative Commons Attribution 4.0 International license (CC-BY 4.0).
