# Supplementary material for: Effectiveness and implementation of a lifestyle modification intervention for women with isolated impaired fasting glucose: Study protocol for a hybrid type 2 study in Kerala, India
Source: Wellcome Open Res. 2022 Feb 15;7:62. [Version 1] doi: 10.12688/wellcomeopenres.17631.1 (PMC9971662; doi:10.12688/wellcomeopenres.17631.1)
Supplement: Peer review history

# Peer review: Reviewer response for version 1

**DOI:** 10.21956/wellcomeopenres.19503.r49435

**Published:** 2023-02-13

**Authors:** Priti Gupta

**Affiliations:** 1 Centre for Chronic Disease Control, New Delhi, Delhi, India

This is a very well written protocol and addresses the importance of primary prevention, where the pre-diabetic who are at risk of developing diabetes in the next five years will be part of a 24-month intervention. There are very few RCTs in this area from India. So the study addresses an important research gap. This study will be carried out in Kerala, which has the highest diabetes prevalence in India. Protocol has elaborated on each and every step of the interventions and how they are going to deliver them. Statistical methods are also well explained. 

 However, few concerns are:

1. Why only women have been selected for trial because this effect the generalizability of this trial results, authors have mentioned it in the limitation but there is no intervention component which is specific for women only
2. Though the authors have stated why they have selected the individual level randomization and not cluster level, as it's a community based intervention chances of contamination will be very high which might dilute the effect of interventions
3. In the control group, they are providing leaflets for lifestyle interventions, people who are diagnosed with diabetes during their assessment should also be provided with these interventions along with referrals to health facilities. 

Is the study design appropriate for the research question?

Yes

Is the rationale for, and objectives of, the study clearly described?

Yes

Are sufficient details of the methods provided to allow replication by others?

Yes

Are the datasets clearly presented in a useable and accessible format?

Not applicable

Reviewer Expertise:

Multimorbidity, implementation research and e-health

I confirm that I have read this submission and believe that I have an appropriate level of expertise to confirm that it is of an acceptable scientific standard.

# Peer review: Reviewer response for version 1

**DOI:** 10.21956/wellcomeopenres.19503.r54040

**Published:** 2023-01-13

**Authors:** David Flood

**Affiliations:** 1 Departments of Medicine, University of Michigan, Ann Arbor, MI, USA

Summary:

 This manuscript presents the study protocol for a type 2 hybrid trial of a lifestyle intervention to achieve normoglycemia among individuals with isolated impaired fasting glucose in Kerala, India. The study is well conceived, and the protocol is very well written. The intervention builds on the well-known Kerala Diabetes Prevention Project (K-DPP). This current protocol is an interesting trial in its focus on individuals with impaired fasting glucose, which is a prediabetes subtype of which there is limited evidence regarding preventative interventions. I look forward to seeing the results in a few years, as this trial will add important evidence on diabetes prevention in low- and middle-income countries where most people with diabetes reside. I have a few comments the authors may wish to consider in manuscript revisions.

 Comments:

1. I wonder if the authors might provide more background and justification on the focus of regression to normoglycemia as the primary outcome rather than new diabetes. I understand that the K-DPP and other trials have been underpowered for incident diabetes among the isolated FBG population. The authors support this decision in the introduction with a citation to the Campbell paper (Campbell MD, et al. Nat Rev Endocrinol 2020;16:395-400), but my reading of that review is that the benefits of regression are primarily observed among the IGT prediabetes phenotype. I see that diabetes incidence is a secondary outcome. Unlike the authors, I am not a prediabetes content expert and I was a bit confused by this choice.
2. The protocol might be strengthened with a justification for a hybrid 2 effectiveness-implementation design. Would the authors consider this trial to be more in line with a hybrid 1? As the authors will be aware, type 1 trials are recommended when interventions have strong face validity and low risk, but there is less evidence for effectiveness in a given context. I may be misreading, but the trial seems to be primarily motivated by the need for evidence on the effectiveness of lifestyle interventions among people with isolated FPG. The protocol has much less detail about the implementation outcomes than the effectiveness outcomes. I did not see a co-primary implementation outcome specified. The individually randomized nature of the trial (rather than cluster RCT) also limits the RE-AIM assessment at the provider/clinic/health system levels. If the authors deem this more in line with a type 2 design, they may wish to add more support for their thinking.
3. Regarding the effect size in the power calculation, for clarity, are the authors using 50% relative risk using "risk" as a desirable outcome? I.e., the RR of the development of normoglycemia would be hypothesized to be 1.5: 18% in control and 27 in intervention (not accounting for contamination)? These values work out when I try to duplicate the sample size calculation, but 1-2 sentences might be helpful for clarity here. Additionally, can the authors cite any effect size estimate beyond their unpublished K-DPP data? 50% RR seems relatively large but, again, I am not an expert in prediabetes like the authors.
4. Would the authors consider including the SPIRIT checklist (below)? I note that most of the checklist elements are included already, but there are a few elements that could be added very briefly.
https://www.equator-network.org/reporting-guidelines/guidelines-for-reporting-outcomes-in-trial-protocols-the-spirit-outcomes-2022-extension/
  Butcher NJ, Monsour A, Mew EJ, et al. Guidelines for Reporting Outcomes in Trial Protocols: The SPIRIT-Outcomes 2022 Extension. JAMA 2022; 328(23): 2345-56.
5. I wanted to praise the authors for their research transparency in sharing about 300 pages of comprehensive study materials on the OSF website. It is nice that we get to see these materials, which also illustrate the immense work the authors have put in to conduct this large community RCT.

Is the study design appropriate for the research question?

Yes

Is the rationale for, and objectives of, the study clearly described?

Yes

Are sufficient details of the methods provided to allow replication by others?

Yes

Are the datasets clearly presented in a useable and accessible format?

Not applicable

Reviewer Expertise:

Diabetes, global health, implementation research, health systems research

I confirm that I have read this submission and believe that I have an appropriate level of expertise to confirm that it is of an acceptable scientific standard.

## References

[1] : Guidelines for Reporting Outcomes in Trial Protocols: The SPIRIT-Outcomes 2022 Extension. *JAMA* .2022;328(23) : 10.1001/jama.2022.21243 2345-2356 10.1001/jama.2022.21243 36512367
